# Correlation analysis of serum miRNA expression levels with the degree of macular edema in patients with retinal vein occlusion and its clinical implications

**DOI:** 10.3389/fneur.2025.1603790

**Published:** 2025-07-22

**Authors:** Jing Lin, Wei Zhao, Hanyi Zang, Xin Bao

**Affiliations:** Ophthalmology Department, Wuxi People’s Hospital Affiliated to Nanjing Medical University, Wuxi, China

**Keywords:** serum miRNA, retinal vein occlusion, macular edema, biomarkers, correlation analysis

## Abstract

**Objective:**

To investigate the correlation between serum microRNA (miRNA) levels and the degree of macular edema in retinal vein occlusion (RVO) patients.

**Methods:**

180 RVO patients were divided into three groups based on macular edema severity: mild, moderate, and severe. Their serum miR-155-5p, miR-17-5p, and miR-375 levels were compared with 60 healthy controls. Pearson correlation analysis assessed the relationship between miRNA levels and clinical outcomes.

**Results:**

miR-155-5p levels were significantly higher in RVO groups compared to controls, while miR-17-5p and miR-375 levels were lower. Increased severity of macular edema correlated with higher miR-155-5p and lower miR-17-5p and miR-375 levels. After treatment, miR-155-5p levels decreased, while miR-17-5p, miR-375, and visual acuity improved. Correlation analysis showed that changes in miR-155-5p were negatively correlated with visual improvement and macular edema resolution, while miR-17-5p and miR-375 were positively correlated with these outcomes.

**Conclusion:**

Serum miR-155-5p, miR-17-5p, and miR-375 levels are associated with macular edema severity in RVO. These miRNAs may serve as biomarkers for disease progression and prognosis, aiding personalized treatment strategies.

## 1 Introduction

Retinal vein occlusion (RVO), a retinal vascular disease that is somewhat prevalent in ophthalmology, has a complex pathogenesis involving multiple factors (1). When retinal veins become occluded, venous return is obstructed, leading to increased intraretinal pressure, and heightened vascular wall permeability. These changes trigger a cascade of pathological events, including retinal hemorrhage, exudation, and macular edema (2). Among these complications, macular edema is one of the most prevalent and visually debilitating manifestations of RVO. It significantly impairs visual function and, in severe cases, may result in irreversible vision loss, substantially diminishing quality of life of individuals (3, 4). The mechanism of macular edema is complex, involving inflammatory responses, increased vascular permeability, and neovascularization. Currently, the diagnosis of macular edema secondary to RVO mainly relies on ophthalmoscopy, optical coherence tomography (OCT), and other imaging examination methods. However, these methods can only diagnose the disease after it occurs and are difficult to achieve early warning and precise assessment (5). Therefore, finding a biomarker that can accurately reflect the degree of macular edema in RVO patients at an early stage is of great significance for improving diagnostic accuracy and guiding treatment strategies.

In the past several years, with the rapid development of molecular biology techniques, microRNA (miRNA), as an important type of non-coding RNA, has been instrumental in the occurrence and development of various diseases (6). The miRNAs regulate the expression of target genes and have a vital part in various biological processes, including cell proliferation, differentiation, and apoptosis, which all have a tight connection to the pathogenesis and progression of numerous ocular diseases (7). Notably, miR-155-5p is a central modulator of immune and inflammatory responses, with elevated expression reported in cardiovascular disorders, autoimmune diseases, and various malignancies, where it influences macrophage activation, vascular inflammation, and tumor microenvironment remodeling (8, 9). miR-17-5p, a member of the miR-17 ~ 92 cluster, is involved in regulating angiogenesis, cell proliferation, and endothelial homeostasis, and has been linked to the progression of lymphomas, colorectal cancer, and atherosclerosis through its effects on vascular and oncogenic signaling pathways (10, 11). miR-375 plays a crucial role in pancreatic *β*-cell function and insulin regulation and has demonstrated tumor-suppressive activity in hepatocellular and gastric cancers (12). These diverse roles highlight their potential relevance as circulating biomarkers in RVO-related macular pathology.

This study investigates the serum expression levels of miR-155-5p, miR-17-5p, and miR-375, aiming to elucidate their correlation with the severity of macular edema in individuals with RVO. Serum samples were collected from RVO individuals, and the expression levels of these miRNAs were measured using real-time fluorescence quantitative PCR (RT-qPCR) and other molecular biology techniques. By integrating these molecular data with clinical parameters and macular edema assessment indicators, this study seeks to uncover the potential mechanistic role of these miRNAs in RVO-associated macular edema, providing insights into their diagnostic and therapeutic implications.

## 2 Materials and methods

### 2.1 Research flowchart

**Figure 1:**
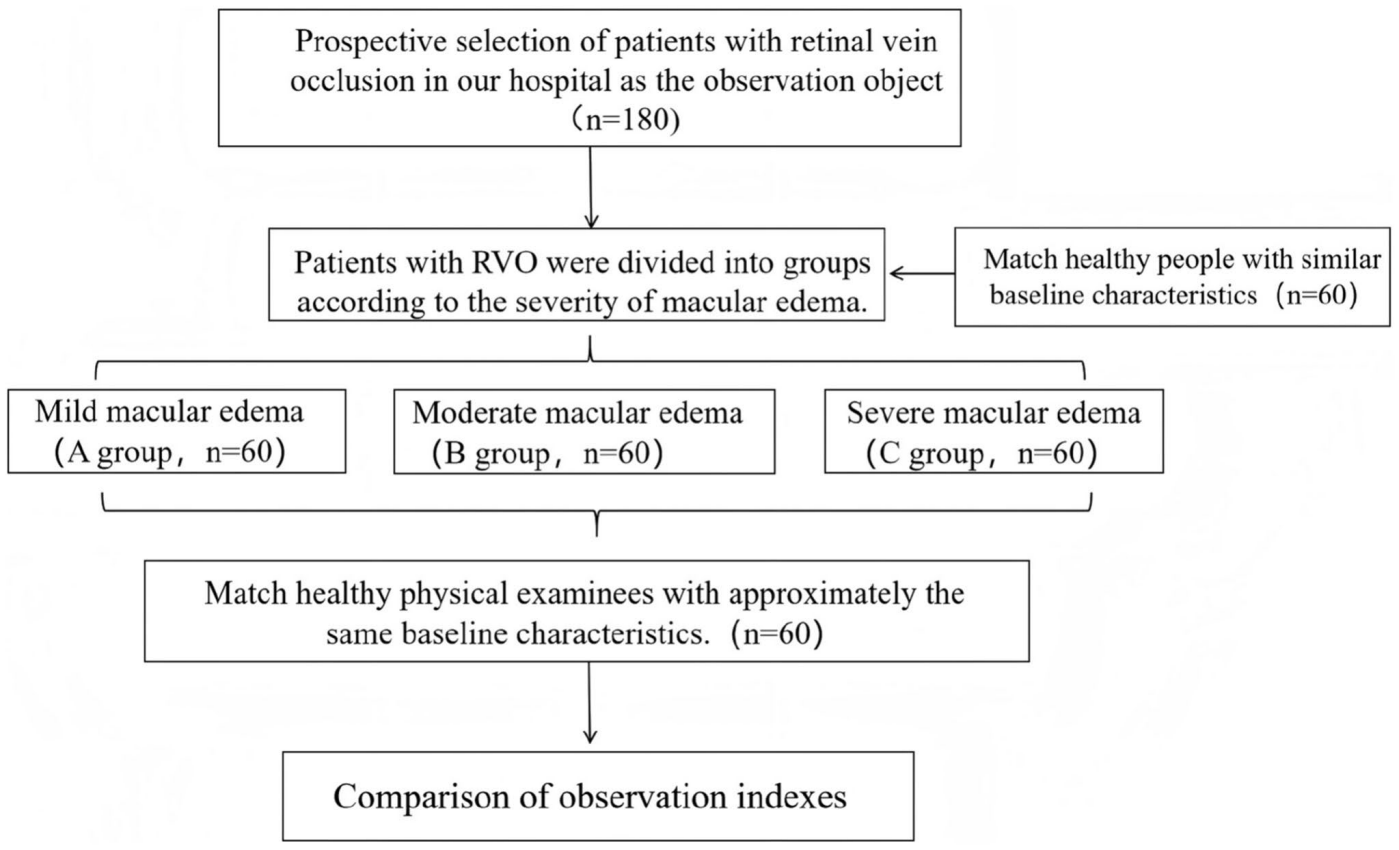
Research flowchart.

Figure 1 shows the flow chart of this research.

### 2.2 Clinical data

A total of 180 individuals with RVO admitted to our hospital from January 2022 to January 2024 were prospectively included as the observation group. According to the severity of their conditions, they were divided into the mild macular edema group (Group A, *n* = 60), the moderate macular edema group (Group B, *n* = 60), and the severe macular edema group (Group C, *n* = 60). Furthermore, 60 people who had health examinations throughout the same time frame were chosen to serve as the control group. Table 1 provides specific information about the patients in each category. Gender, age, body mass index (BMI), smoking and drinking histories did not differ statistically significantly across the groups (*p* > 0.05). Our hospital’s medical ethics committee authorized this study, and each patient completed an informed consent form. The assessment criteria for the degree of macular edema: The assessment of macular edema mainly relies on optical coherence tomography (OCT). OCT can offer high-resolution cross-sectional images and accurately measure the central foveal thickness (CFT) and volume changes in the macular area. CFT measurement: mild edema: CFT < 300 μm; moderate edema: CFT = 300–400 μm; severe edema: CFT > 400 μm. OCT image features: mild edema: slight foveal thickening with no apparent cystoid changes; moderate: Noticeable foveal thickening with mild cystoid changes; severe: significant foveal thickening accompanied by prominent cystoid changes and disruption of retinal structural.

**Table 1:** Baseline characteristics comparison ( $\bar{x}$ ± s).

Characteristic	Healthy control group (*n* = 60)	Mild macular edema (*n* = 60)	Moderate macular edema (*n* = 60)	Severe macular edema (*n* = 60)	*χ^2^/F*-value	*p*-value
Age (years, $\bar{x}$ ± s)	60.25 ± 10.71	60.41 ± 10.34	61.37 ± 9.97	60.95 ± 10.22	0.149	0.931
Gender (Male/%)	30 (50.00)	32 (53.33)	29 (48.33)	31 (51.67)	1.079	0.782
BMI (kg/m^2^, $\bar{x}$ ± s)	24.18 ± 3.44	25.03 ± 4.18	24.66 ± 3.56	24.81 ± 4.04	0.534	0.659
History of smoking (Yes/%)	18 (30.00)	15 (25.00)	19 (31.67)	13 (21.67)	1.920	0.589
History of alcohol consumption (Yes/%)	9 (15.00)	11 (18.33)	12 (20.00)	7 (11.67)	1.806	0.614
Hypertension (Yes/%)	–	5 (87.33)	7 (11.67)	6 (10.00)	0.370	0.831
Cardiovascular diseases (Yes/%)	–	8 (13.33)	6 (10.00)	8 (13.33)	0.414	0.813
Diabetes (Yes/%)	–	11 (18.33)	12 (20.00)	10 (16.67)	0.260	0.878
RVO type (*n*/%)					0.327	0.849
CRVO	–	22 (36.67)	25 (41.67)	23 (38.33)		
BRVO	–	38 (63.33)	35 (58.33)	37 (61.67)		
Classification of Edema (*n*/%)					0.703	0.704
Simple type	–	15 (25.00)	18 (30.00)	19 (31.67)		
Compound type	–	45 (75.00)	42 (70.00)	41 (68.33)		
Duration of illness (month, $\bar{x}$ ± s)	–	6.41 ± 3.05	6.33 ± 3.12	6.54 ± 3.21	0.069	0.933
Fasting blood glucose (mmol/L, $\bar{x}$ ± s)	4.51 ± 1.88	6.33 ± 1.74	6.47 ± 1.95	6.51 ± 1.47	17.896	<0.001
TC (mmol/L, $\bar{x}$ ± s)	4.06 ± 1.23	4.46 ± 0.58	5.61 ± 1.77	5.45 ± 1.83	16.379	<0.001
LDL-C (mmol/L, $\bar{x}$ ± s)	2.83 ± 0.17	3.69 ± 0.54	3.55 ± 0.47	3.77 ± 0.81	37.010	<0.001
ALT exception (Yes, %)	–	5 (8.33)	4 (6.67)	6 (10.00)	0.436	0.804
AST exception (Yes, %)	–	3 (5.00)	1 (1.67)	4 (6.67)	1.831	0.400
Cr exception (Yes, %)	–	5 (8.33)	3 (5.00)	4 (6.67)	0.775	0.679
BUN abnormal (Yes, %)	–	3 (5.00)	4 (6.67)	3 (5.00)	0.212	0.900

Inclusion criteria: (1) Diagnosed with RVO according to the *EURETINA Guidelines for the Diagnosis and Treatment of RVO* (13); (2) Unilateral involvement; (3) First-time diagnosis of RVO; (4) No prior relevant treatment before hospital admission; (5) Age ≥ 18 years.

Exclusion criteria: (1) Presence of diabetic retinopathy or hypertensive retinopathy; (2) Concurrent ocular diseases, including glaucoma, elevated intraocular pressure, high myopia, epiretinal membrane, vitreomacular traction syndrome, retinal detachment, severe refractive media opacity, or a history of ocular trauma; (3) History of cataract surgery or prior vitreoretinal treatments, such as vitrectomy, intravitreal injections, or retinal laser photocoagulation; (4) Poor compliance or inability to cooperate with study procedures; (5) Incomplete medical records; (6) Pregnancy or lactation.

### 2.3 General information collection

Detailed interviews were conducted and recorded for all the included subjects, including their names, ages, and genders, disease duration, past medical history, disease treatment history, and overall health conditions. Relevant tests were completed fasting blood glucose, total cholesterol (TC), Blood urea nitrogen (BUN), creatinine (Cr), aspartate aminotransferase (AST), alanine aminotransferase (ALT), and low-density lipoprotein cholesterol (LDL-C). All individuals underwent intraocular pressure measurement, visual acuity measurement, slit-lamp examination after mydriasis (including anterior segment inflammation, anterior chamber depth, and lens opacity), and completed ophthalmic examinations such as B-ultrasound of both eyes, color fundus photography, OCT, and fundus fluorescein angiography (FFA). The current study was approved by the Ethics Committee of the Wuxi People’s Hospital Affiliated to Nanjing Medical University (approval number WPH202411202). Written informed consents from all patients were obtained in any experimental work with humans.

### 2.4 Treatment protocol

All patients with RVO and macular edema received standardized intravitreal injections of ranibizumab (0.5 mg/0.05 mL) monthly for 6 months. Treatment was initiated at baseline, with follow-up assessments at 3 and 6 months using OCT and best-corrected visual acuity (BCVA). No corticosteroids or laser photocoagulation were used, ensuring uniform treatment across all groups (mild, moderate, severe). Injections were performed under sterile conditions by experienced ophthalmologists.

### 2.5 Optical coherence tomography imaging

All patients with RVO who were included in the study and required intravitreal injection underwent OCT examination to measure the central macular thickness (CMT) value before the operation. For patients with vitreous hemorrhage who were diagnosed with RVO after the operation, the CMT value was measured on the second day after the operation. Before the examination, patients were provided with detailed instructions regarding the procedure and its precautions. They were assisted in assuming a comfortable seated position, with their chin placed gently in the groove and their forehead securely positioned against the headrest. Both eyes were instructed to maintain a straight gaze. The examiner then proceeded with macular scanning, specifically performing linear scans in two directions: horizontally (from left to right) and vertically (from top to bottom), focusing on the fovea centralis. Each scan had a length of 4 to 5 mm. Following the scan, the CFT was measured using Spectral-domain OCT (Spectralis, Heidelberg Engineering, Heidelberg, Germany), described as the separation between the retinal pigment epithelium’s basal membrane and inner limiting membrane. This measurement was used to assess the severity of macular edema. The CFT values were obtained by averaging three measurements made by two experienced ophthalmologists and recorded accordingly.

### 2.6 Fundus fluorescein angiography assessment

All RVO patients included in the study underwent FFA examination using a confocal scanning laser ophthalmoscope (e.g., Zeiss FF450 Plus). Before the examination, the patient’s information was carefully checked, and their vital signs and liver and kidney functions were examined. There were no systemic adverse conditions, such as no history of drug allergy, no cerebral infarction or myocardial infarction, etc. The risks and precautions were informed to the patients and their families, and the relevant consent forms were signed. The pupils were completely dilated with compound tropicamide eye drops. After dilation, fundus photography of both eyes was performed first, followed by FFA examination. The examination was conducted by the same technician. The specific procedure for the FFA examination was as follows: The patient was assisted into a comfortable seated position, with their chin gently placed in the groove, forehead pressed against the headrest, and arms resting on the table for easy access and exposure. Initially, an allergy test was performed by injecting a diluted sodium fluorescein solution into the antecubital vein to check for any allergic reactions. Once it was confirmed that there was no reaction, a 5 mL (10%) sodium fluorescein injection was administered. The timing of the injection was recorded, and fundus images were captured at 30-s intervals for 5 min, followed by late-phase imaging at 10 min in different quadrants of the retina. Finally, two experienced ophthalmologists independently reviewed the images, making the necessary observations and relevant clinical records.

### 2.7 Quantification of serum miRNAs by qRT-PCR

An RNA extraction kit (Aldrich, United States) was used to obtain total RNA. A High-Capacity cDNA Reverse Transcription Kit (Applied Biosystems, United States) was used to reverse transcribe the isolated RNA into cDNA. SYBR Green dye (Applied Biosystems, USA) and RNA samples were used in the qRT-PCR experiments. The following is how the reaction mixture was made: (1) 0.4 μL ROX Reference Dye II; (2) 0.2 μL forward primer; (3) 0.2 μL reverse primer; (4) 6.0 μL ddH₂O; (5) 10 μL SYBR Premix Ex Taq II. The following were the amplification conditions: 90 s at 95°C, then 40 cycles of 30 s at 95°C, 30 s at 63°C, and 30 s at 72°C. Table 2 lists the primer sequences for U6, which served as the internal reference gene. Every sample was performed three times, and the analysis was based on the average value. The 2-ΔΔCt technique was used to determine the relative expression levels of the target genes miR-155-5p, miR-17-5p, and miR-375 (where Ct is the cycle threshold).

**Table 2:** Primer sequence list.

Gene		Sequences
miR-155-5p	Upstream primer	5’-GTAACCCGTTGAACCCCATT-3’
	Downstream primer	5’-CCATCCAATCGGTAGTAGCG-3’
miR-17-5p	Upstream primer	5’-CCAGGATCCTTTATAFTTFTTAFAFTTTG-3’
	Downstream primer	5’-CGGAATTCTAATCTACTTCACTATCTGCA-3’
miR-375	Upstream primer	5’-TTTGTTCGTTCGGCTCGC-3’
	Downstream primer	5’-CGCTTCGGCAGCACATATAC-3’

### 2.8 Statistical analysis

The statistical program SPSS 21.0 was utilized. All measurement data were tested for homogeneity of variance and normal distribution before statistical analysis to make sure they satisfied the criteria for a normal distribution or an approximation normal distribution. $\bar{x}$ ± s was used to express the data. Repeated measures ANOVA was used to examine data from repeated measurements. The t-test was used to compare the two groups. The *χ*^2^ test was used to assess the count data, which were reported as *n* (%). For pre- and post-treatment comparisons of serum miRNA levels and BCVA, data from all RVO patients (Groups A, B, and C) were pooled to evaluate overall treatment effects. Pearson correlation analysis was used to assess relationships between changes in miRNA levels and CFT with a two-sided *p*-value < 0.05 considered significant.

## 3 Results

### 3.1 Baseline data

The baseline data of each group of patients were compared. General data, including age, gender, BMI, history of drinking and smoking, did not show statistically significant differences (*p* > 0.05). Groups A, B, and C had higher fasting blood glucose, TC, and LDL-C values than the healthy control group; these differences were statistically significant (*p* < 0.05). The kinds of RVO, edema categorization, illness duration, and aberrant proportions of ALT, AST, Cr, and BUN did not differ statistically significantly between groups A, B, and C (*p* > 0.05; Table 1).

### 3.2 Comparison of serum miR-155-5p, miR-17-5p, miR-375 levels and BCVA values among patients with different degrees of macular edema

**Figure 2:**
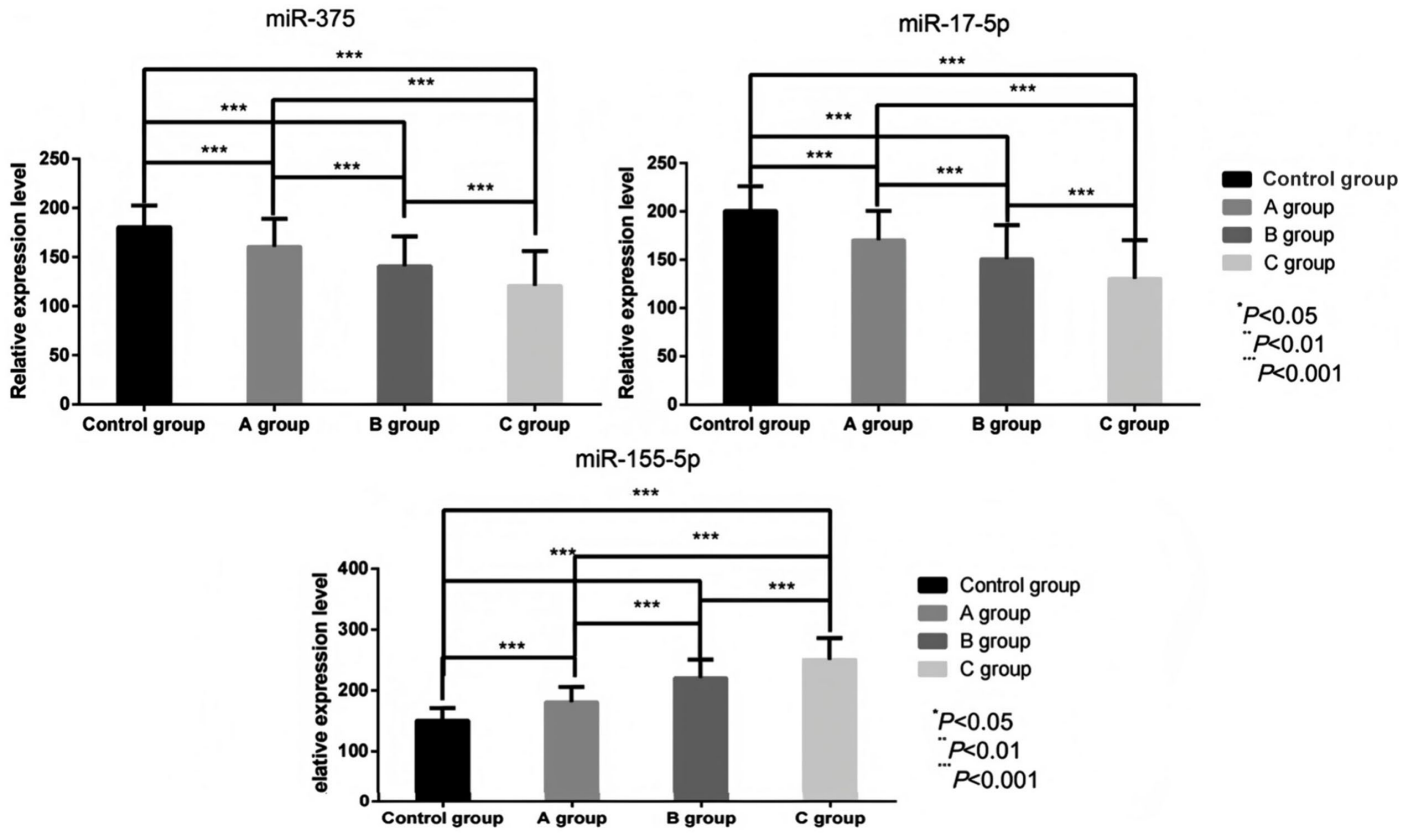
Comparison of serum miR-155-5p, miR-17-5p, and miR-375 levels before treatment in individuals with different degrees of macular edema.

**Figure 3:**
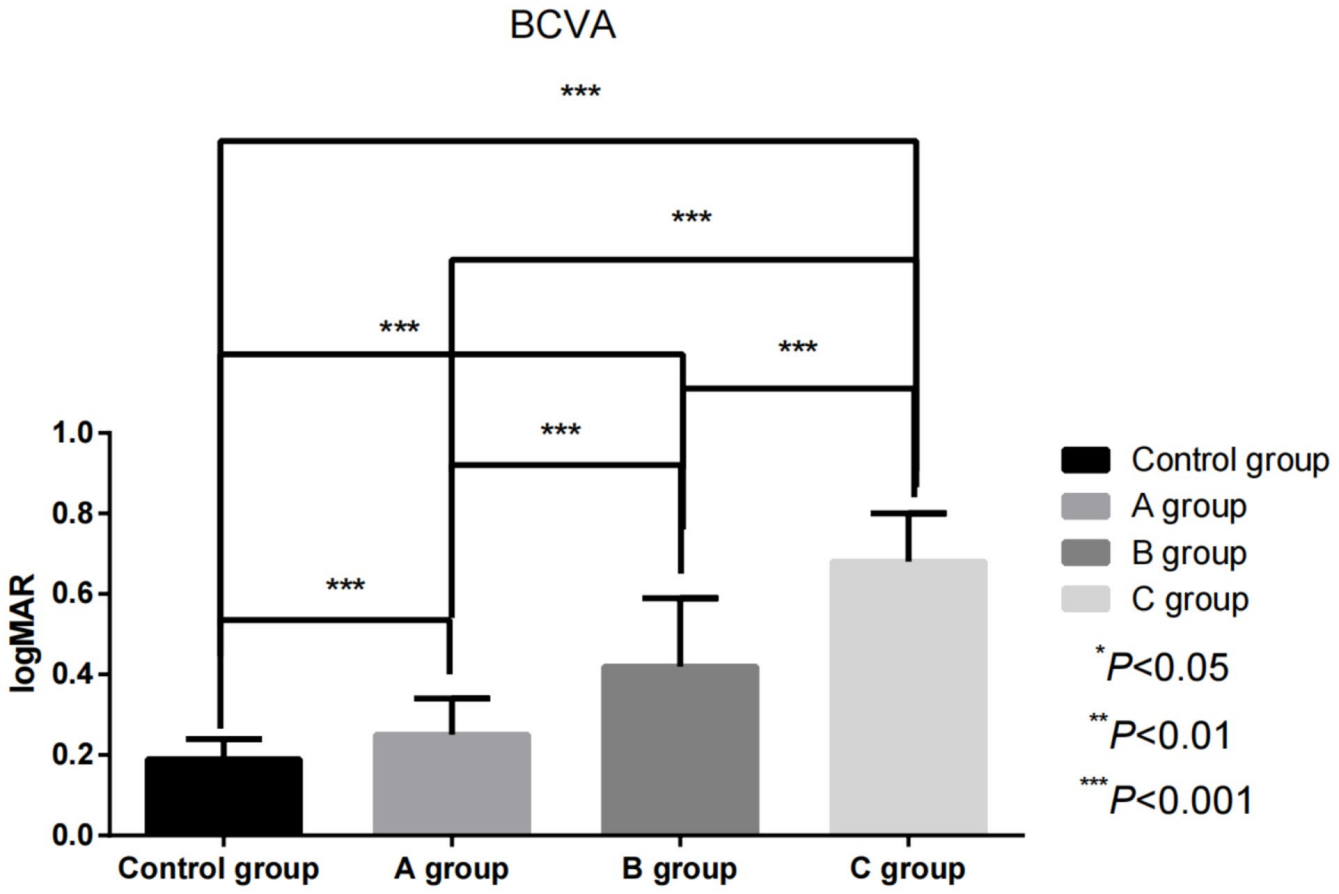
Comparison of BCVA values among individuals with different degrees of macular edema before treatment.

The serum levels of miR-155-5p in Group A, Group B, and Group C were considerably higher than those in the control group (180.71 ± 25.03) vs. (150.36 ± 20.64), (220.41 ± 30.54) vs. (150.36 ± 20.64), (250.36 ± 35.82) vs. (150.36 ± 20.64; *t* = 7.246, 14.720, 18.737; *p* < 0.05). Conversely, the levels of miR-17-5p (170.35 ± 30.14) vs. (200.54 ± 25.62), (150.65 ± 35.32) vs. (200.54 ± 25.62), (130.24 ± 40.06) vs. (200.54 ± 25.62; *t* = 5.912, 8.857, 11.452; *p* < 0.05), miR-375 (160.51 ± 28.57) vs. (180.53 ± 22.16), (140.73 ± 30.38) vs. (180.53 ± 22.16), (120.68 ± 35.38) vs. (180.53 ± 22.16; *t* = 4.289, 8.198, 11.105; *p* < 0.05) were considerably lower than those in the control group (*p* < 0.05). BCVA demonstrated significant impairment in RVO patients, with values of 0.25 ± 0.09 in Group A versus control 0.19 ± 0.05 (*t* = 9.678, *p* < 0.05), 0.42 ± 0.17 in Group B (*t* = 22.205, *p* < 0.05), and 0.68 ± 0.12 in Group C (*t* = 29.196, *p* < 0.05). As macular edema severity increased, miR-155-5p exhibited a progressive upward trend while miR-17-5p, miR-375, and visual function declined. These intergroup differences were statistically significant (miR-155-5p: *F* = 141.679; miR-17-5p: *F* = 48.702; miR-375: *F* = 45.644; BCVA: *F* = 212.886; all *p* < 0.05), as visually represented in Figures 2, 3.

### 3.3 Serum miR-155-5p, miR-17-5p, miR-375 and BCVA changes in all RVO macular edema after treatment

**Figure 4:**
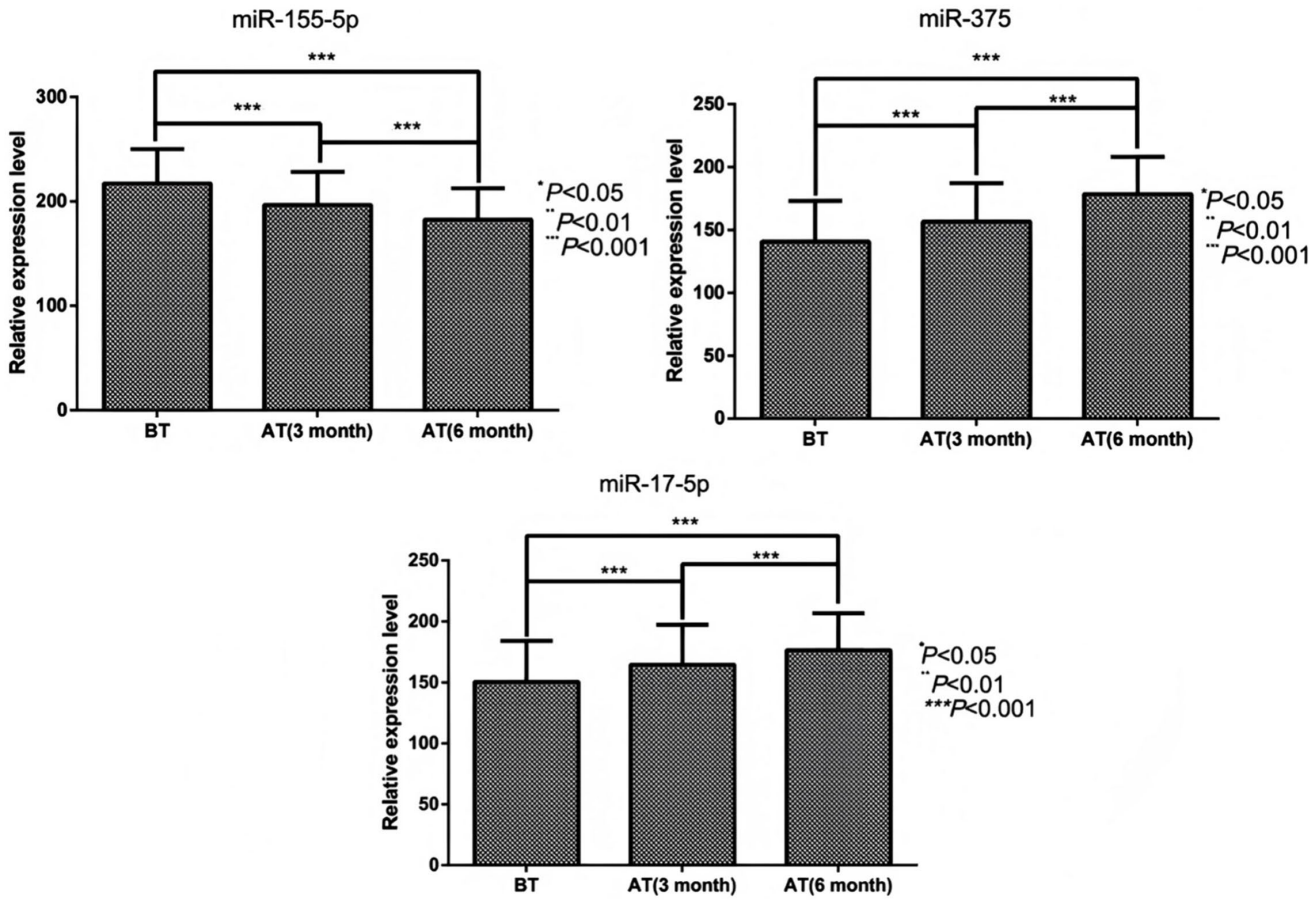
Comparison of serum miR-155-5p, miR-17-5p, and miR-375 levels before and after treatment in pooled RVO patients. For miR-155-5p: comparison between groups, *F* = 30.362, *p* < 0.001; comparison at different time points, *F* = 54.717, *p* < 0.001; group × time interaction, *F* = 86.241, *p* < 0.001. For miR-17-5p: comparison between groups, *F* = 18.641, *p* < 0.001; comparison at different time points, *F* = 29.517, *p* < 0.001; group × time interaction, *F* = 44.205, *p* < 0.001. For miR-375: comparison between groups, *F* = 13.352, *p* < 0.001; comparison at different time points, *F* = 68.016, p < 0.001; group × time interaction, *F* = 112.542, *p* < 0.001. Before treatment (BT); 3 months after treatment (AT 3 month); 6 months after treatment (AT 6 month).

**Figure 5:**
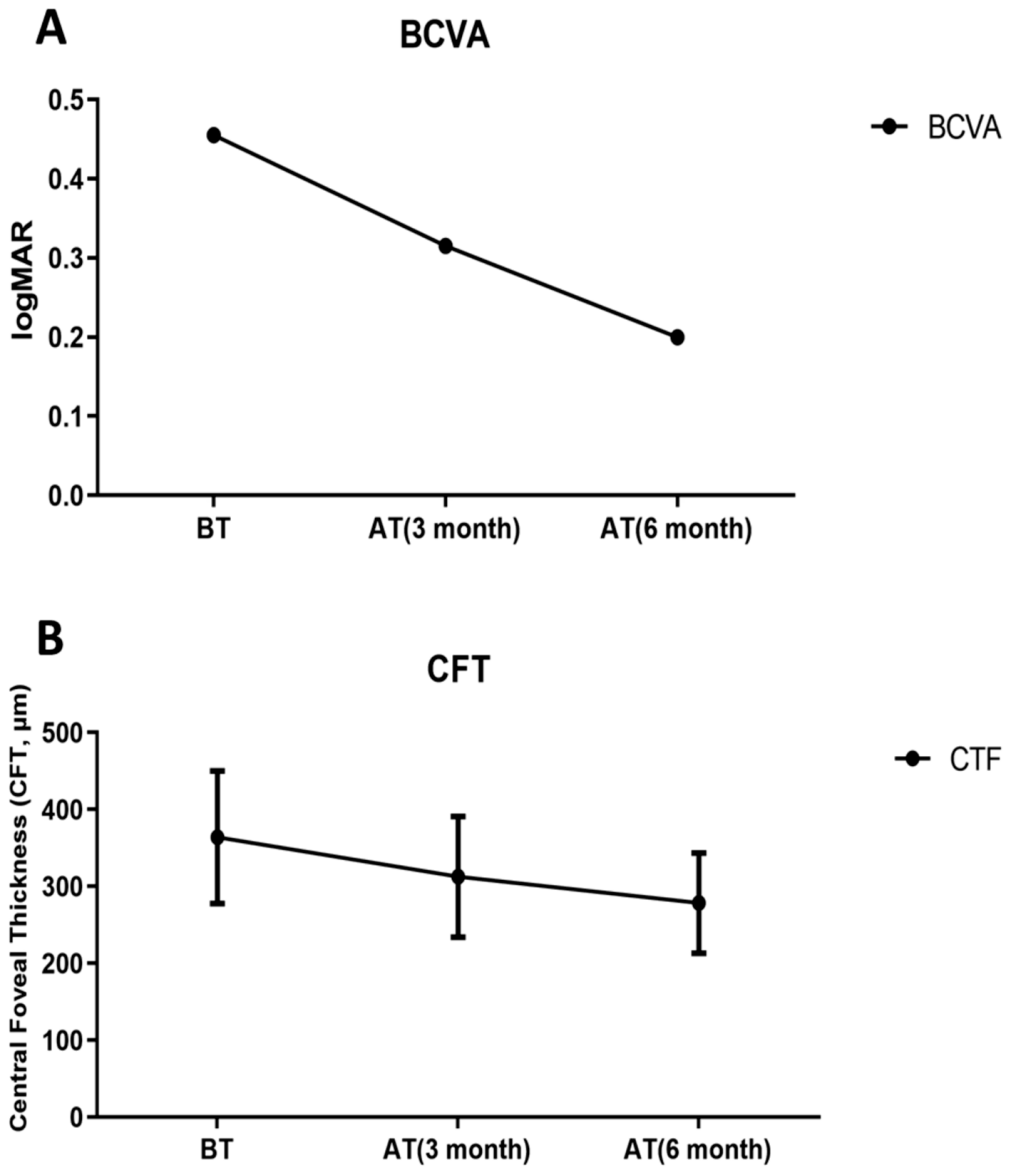
Post-treatment changes in pooled retinal vein occlusion (RVO) patients. **(A)** Best-corrected visual acuity (BCVA), Significant time effect: *F* = 109.529, *p* < 0.001; **(B)** Central foveal thickness (CFT), Significant time effect: *F* = 214.519, *p* < 0.001. Before treatment (BT); 3 months after treatment (AT 3 month); 6 months after treatment (AT 6 month).

Compared with the pre-treatment values, the pooled serum data from all RVO macular edema patients (Groups A, B, and C combined) showed that, the serum miR-155-5p levels of RVO macular edema patients were significantly decreased after 3 months and 6 months of treatment (*t* = 3.463, 6.004, *p* < 0.05), while the levels of miR-17-5p (*t* = 2.334, 4.471, *p* < 0.05), miR-375 (*t* = 2.783, 6.668, *p* < 0.05) increased. Visual acuity demonstrated significant recovery, with BCVA improvements at 3 months (*t* = 2.552, *p* < 0.05) and 6 months (*t* = 8.754, *p* < 0.05). Structural improvements accompanied these changes, as CFT values significantly decreased from 363.8 ± 86.3 μm at baseline to 312.6 ± 78.5 μm at 3 months (*p* < 0.05) and to 278.4 ± 65.2 μm at 6 months (*p* < 0.05). This indicates that the regulatory roles of miR-17-5p and miR-375 in vascular homeostasis and apoptosis are enhanced (Figures 4, 5).

### 3.4 Correlation analysis of miRNA and clinical efficacy

The expression changes of miR-155-5p had a negative correlation with the reduction of CFT and the improvement of visual acuity (*r* = −0.364, −0.418, *p* < 0.05), indicating that the decrease in miR-155-5p expression was associated with the alleviation of macular edema and the enhancement of visual acuity; the expression changes of miR-17-5p and miR-375 had a positive relationship with the reduction of CFT and the improvement of visual acuity (*r* = 0.537, 0.389, *p* < 0.05; r = 0.418, 0.605, *p* < 0.05), suggesting that the increase in the expression of these miRNAs was related to the alleviation of macular edema and the improvement of visual acuity (Table 3).

**Table 3:** Results of correlation analysis.

Group	CFT		BCVA	
	*r*	*P*	*r*	*P*
miR-155-5p	−0.364	0.031	−0.418	0.004
miR-17-5p	0.537	<0.001	0.389	<0.001
miR-375	0.418	<0.001	0.605	<0.001

## 4 Discussion

RVO occurs often in retinal vascular disease. Epidemiological characteristics show that it is particularly prevalent among the elderly, especially those over 60 years old (14). According to epidemiological research data, the annual incidence rate of RVO varies. In a study of individuals aged 30 years and older (15), the overall incidence rate of all RVO was 5.2 per 1,000 individuals, with branch retinal vein occlusion (BRVO) occurring at a rate of 4.42 per 1,000, and central retinal vein occlusion (CRVO) at a rate of 0.8 per 1,000. Additionally, other studies have stated that the 5-year incidence rate of RVO ranges from 0.6 to 1.2%. However, baseline incidence rates over 10 or 15 years vary depending on regional and population-specific factors (16). CRVO is typically associated with more severe vision loss, whereas BRVO often leads to localized vision impairment. The clinical significance of RVO extends beyond its direct impact on vision, as it also increases the risk of systemic vascular diseases, including hypertension, diabetes, and cardiovascular diseases. Given these potential complications, early diagnosis, and prompt treatment of RVO are crucial for preventing further vision deterioration and reducing the risk of associated systemic health issues.

With the quick advancement of molecular biology methods in recent years, more research has started to examine the connection between serum miRNA expression levels and RVO and associated consequences. MiRNAs are a kind of non-coding RNA that may control post-transcriptional gene expression and are implicated in a number of biological processes, including as angiogenesis, differentiation, apoptosis, and cell proliferation (17, 18). Research has demonstrated that miR-155-5p regulates the oxidative stress-induced apoptosis of retinal pigment epithelium cells (19). Additionally, the expression level of miR-375 in patients with diabetic macular edema (DME) is negatively correlated with the degree of macular edema, implying that it might contribute to the pathophysiology of macular edema (20). By detecting the levels of these miRNAs in serum, not only can new biomarkers for the diagnosis of RVO be provided, but also the progression and prognosis of patients can be evaluated, and individualized treatment plans can be guided. However, few studies are available at the moment on the expression levels of miR-155-5p, miR-175-5p, and miR-375 in RVO-ME and their correlation with the degree of macular edema. In this study, it was discovered that the expression level of miR-155-5p in the serum of RVO patients demonstrated a noteworthy increasing trend with the aggravation of macular edema. Compared with the healthy control group, the expression levels of miR-155-5p in the mild, moderate, and severe macular edema groups were all significantly increased. This difference may be due to the regulatory role of miR-155-5p in inflammatory responses and angiogenesis. Macular edema caused by RVO is often accompanied by intraocular inflammatory responses and increased vascular permeability, and the upregulation of miR-155-5p, as an inflammation-related microRNA, may reflect the intensification of intraocular inflammatory responses (21). Furthermore, by controlling the expression of genes linked to angiogenesis, miR-155-5p may possibly have a role in the onset and progression of macular edema. In contrast to miR-155-5p, the expression level of miR-17-5p in RVO patients significantly decreased as the severity of macular edema increased. This finding may suggest that miR-17-5p plays a critical protective role in safeguarding the retina from edema-induced damage. The correct function and structural integrity of retinal cells depend on the regulation of critical processes including oxidative stress and apoptosis, which miR-17-5p is implicated in. These results highlight the potential of miR-17-5p as a therapeutic target for preventing or mitigating retinal damage in RVO (22, 23). In macular edema caused by RVO, retinal cells may be threatened by oxidative stress and apoptosis, leading to the downregulation of miR-17-5p. This downregulation may reflect the impairment of the self-protection mechanism of retinal cells in response to injury. Similar to miR-17-5p, the serum expression level of miR-375 in RVO patients also significantly decreased as the severity of macular edema worsened. miR-375 is crucial for retinal development and the maintenance of retinal function, and its downregulation may be associated with functional impairment of retinal cells. This suggests that miR-375 plays a significant role in preserving retinal health, and its reduction could contribute to the progression of retinal dysfunction in RVO (24, 25). In macular edema caused by RVO, retinal cells can suffer damage due to ischemia, hypoxia, and inflammatory responses, which may lead to a decrease in miR-375 expression. This reduction in miR-375 could further exacerbate the damage and dysfunction of retinal cells, contributing to the progression of macular edema and worsening visual impairment.

Further analysis revealed that the BCVA values of RVO patients were significantly associated with the levels of expression of miR-155-5p, miR-175-5p, and miR-375 in the serum. The BCVA values likewise exhibited a negative trend when the expression levels of miR-155-5p and miR-175-5p and miR-375 declined, respectively. This indicates that the expression levels of these microRNAs may serve as potential biomarkers for assessing the degree of macular edema and visual prognosis in RVO patients. The high expression of miR-155-5p may reflect an intensified intraocular inflammatory response and increased vascular permeability, while the low expression of miR-175-5p and miR-375 may suggest impaired retinal cell function and compromised self-protection mechanisms. These factors collectively contribute to the decline in vision in RVO patients. From a clinical perspective, the findings of this investigation offer new insights for the diagnosis and treatment of RVO patients. On one hand, by detecting the expression levels of microRNAs in the serum, clinicians can more accurately assess the severity of macular edema and visual impairment in RVO patients, leading to the formulation of more personalized treatment plans. On the other hand, these microRNAs show potential as novel biomarkers for the early screening and disease monitoring of RVO.

However, this study has several limitations. As an observational study, it cannot establish a causal relationship between changes in the expression levels of miR-155-5p, miR-17-5p, and miR-375 and the progression of macular edema or visual improvement. Additionally, the study did not explore the specific mechanisms through which these microRNAs operate, relying on existing literature to make inferences. Furthermore, the relationship between the expression changes of these microRNAs and the prognosis of RVO individuals remains unexplored. Future research is essential to further validate the role of these microRNAs in RVO-induced macular edema at the molecular mechanism level. In-depth studies of their mechanisms will result in a deeper comprehension of how these microRNAs regulate intraocular inflammatory responses, angiogenesis, and retinal cell function, potentially revealing new therapeutic targets and strategies for RVO treatment. Additionally, larger-scale cohort studies and long-term follow-up observations are necessary to further validate the reliability and clinical applicability of these microRNAs as biomarkers, providing more accurate tools for the early screening, disease monitoring, and prognosis assessment of RVO.

## 5 Conclusion

In conclusion, the expression levels of miR-155-5p, miR-175-5p, and miR-375 in serum are correlated with the degree of macular edema in RVO patients. These microRNAs may serve as potential biomarkers for evaluating the degree of macular edema and visual prognosis in RVO patients, and provide new insights for clinical diagnosis and treatment.

## Data Availability

The raw data supporting the conclusions of this article will be made available by the authors, without undue reservation.
